# Evaluation of Subclinical Extension of Basal Cell Carcinoma

**Published:** 2017-09

**Authors:** Arash Beiraghi Toosi, Nema Mohamadian Roshan, Mahdi Ghoncheh

**Affiliations:** 1 Endoscopic and Minimally Invasive Surgery Research Center, Department of Plastic Surgery, Mashhad University of Medical Sciences, Mashhad, Iran;; 2 Department of Pathology, Mashhad University of Medical Sciences, Mashhad, Iran

**Keywords:** Basal cell carcinoma, Subclinical, Type, Margin

## Abstract

**BACKGROUND:**

Basal cell carcinoma (BCC) is the most common malignancy with increasing incidence worldwide. The tumor invades surrounding tissues in an irregular pattern via subclinical and microscopic finger-like growths known as subclinical extension. Subclinical extension may be responsible for incomplete resection of the tumor. This study investigates the subclinical extension of BCC.

**METHODS:**

In a retrospective study for evaluation of subclinical extension of BCC, Patients’ demographic data and characteristics (disease duration, location, size, and history of radiotherapy) were documented. Pathology samples were assessed in terms of histological type, subclinical extension, depth, and involvement of margins.

**RESULTS:**

The study was conducted on 102 pathological samples of 84 patients (49 males, 35 females) with BCC. The mean age was 65.4±12.55 years. Overall, 83% of pathology samples had subclinical extension. Subclinical extension had no correlation with lesion size (*p*=0.591; r=0.056), but had a direct correlation with lesion depth (*p*=0.033; r=0.220). Resection of the tumor with a margin of 5.5 mm eliminated the entire lesion and its subclinical extension area with a confidence rate of 95%.

**CONCLUSION:**

Based on this study, resection of BCC lesions with a margin of 5.5 mm will eradicate the whole lesion including the subclinical extension area with 95% confidence rate. Depth of the tumor, not its size or histologic subtype, affects the required margin of excision.

## INTRODUCTION

Basal cell carcinoma (BCC) is the most common malignancy with increasing incidence worldwide.^1^^,^^2^ It is more common in the fifth and sixth decades of life.^3^^-^^5^ The tumor is locally invasive and it can produce significant morbidity for the patient. The tumor invades surrounding tissues in an irregular pattern via subclinical and microscopic finger-like growths.^6^ These appendages, growing beyond the visible margins of the main tumor,^7^^-^^9^ are known as subclinical extension. Surgical excision is the main treatment of BCC among many alternative therapies.^10^ However, incomplete excision may be a problem even with surgical excision.^11^^-^^13^ It may be due to subclinical extension of the tumor beyond the visible clinical margins.^8^ Although Mohs micrographic surgery (MMS) can control margins precisely and decrease the probability of the remaining tumor,^14^ this modality is not available in all centers. After resection of the tumor with MMS, there is still 1-2% chance of recurrence and it may be due to subclinical extension of BCC.^15^ Accurate estimation of subclinical extension can reduce morbidity of surgery and risk of recurrence. In this study, subclinical extension of BCC was evaluated.

## MATERIALS AND METHODS

Patients with BCC lesions operated by the first author during 2009 to 2013 were enrolled in the study. In our center, due to lack of access to MOHS surgery, all BCC lesions were routinely removed with 5 mm safe margins. Cases with concurrent cancer, patients who had history of surgery near to the location of the lesion, recurrent lesions, and basosquamous cases were excluded. Those with more than one lesion were considered as separate cases.

Demographic data (age and gender), as well as information related to the disease (disease duration, location and size of the lesion, and history of radiotherapy) were recorded in the check list. *Slides prepared from paraffin tissue blocks* were examined in terms of histological type, depth of invasion, subclinical extension, and margin involvement. The microscopic extension of the lesions under the epidermis beyond the visible surface involvement were measured at 3, 6, 9 and 12 o’clock directions by a pathologist. The largest extension was considered as the subclinical extension.

Coded data were analyzed using SPSS software for Windows (Version 16, SPSS Inc., Chicago, IL, USA). The qualitative variables were compared by Chi-Square test. Comparisons of the depth or size of the lesions in the both genders were performed using the Mann-Whitney test. The depth and size of lesions in different histological types were compared using one-way ANOVA test and Tukey post-hoc test. The relationship between the subclinical extension and lesion size was evaluated with Pearson test and regression graph was shown. Significance level was considered more than 95%.

## RESULTS

The study was conducted on 102 *paraffin tissue blocks* of 84 patients (49 men and 35 women). Out of 102 pathology cases, 65 were belonging to male patients (63.7%) and 37 to females (36.3%). The mean age of the patients was 65.4±12.55 years. The mean duration of the disease was 2.5±2.48 years. The most common site of the lesions was nose (31.2%), followed by scalp (16.1%), lower eyelid (11.8%), cheeks (10.8%), forehead (8.6%), and lower lip (5.4%). Other parts of the body comprised 16.1% of cases.

The most common histological types were nodular (83.6%), adenoid (9.1%), and superficial types (7.1%). Other less common types (4%) included morphoeic and metatypical (Table 1). The Tukey post-hoc test showed that the prevalence of superficial lesions was significantly less than nodular (*p*<0.001) and Adenoid types (*p*=0.008) and no significant difference was seen between the other types. A statistically significant direct correlation was observed between the size of the lesion and the depth of the lesion (*p*=0.001, r=0.621).

**Table 1:** Comparison of the mean size, depth, and subclinical extension (in millimeters) in different histological types of BCC

**Type**	**Nodular**	**Adenoid**	**Superficial**	**Morphoeic**	**Metatypical** ^1^	***p*** **value**
Size (mean±SD)	8.7±6.19	11.0±7.23	2.7±1.60	8.0±0.57	6.0	0.093**
Depth (mean±SD)	3.8±2.35	5.3±1.83	0.9±.56	4.7±0.50	3.0	0.001**
Subclinical Extension (mean±SD)	2.1±1.92	2.1±2.84	0.9±1.03	1.0±1.41	2.0	0.554**

1: one case had this type of BCC,

** : ANOVA test

**Fig. 1:**
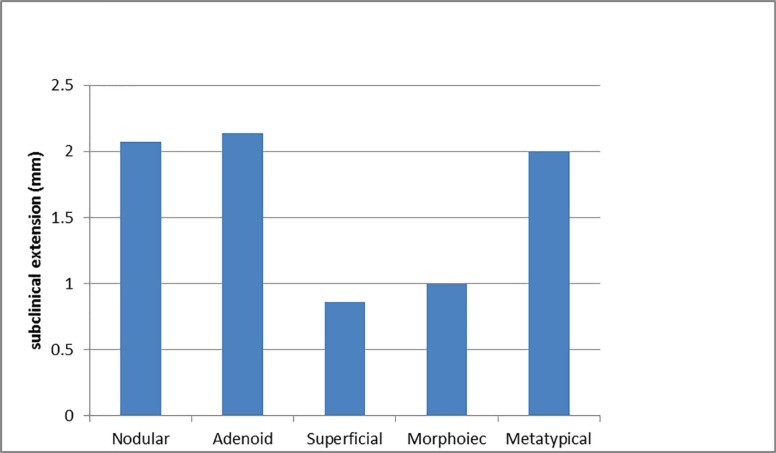
The mean extent of subclinical extension in various histologic types of BCC

Eight cases (7.8%) had positive margin involvement. These cases were excluded from the evaluation of subclinical extension. Of the remaining 94 cases, 83.0% had subclinical extension while 17.0% had no subclinical extension in any of the 4 directions (3, 6, 9, or 12 o’clock). The mean extent of subclinical extension was 1.9±1.98 mm (0.0 to 10.5 mm). Subclinical extension was not correlated with the gender (*p*=0.719), the age of the patients (*p*=0.366), the disease duration (*p*=0.297, r=0.109), the size of the lesions (*p*=0.591, r=0.056), the location of the lesions (*p*=0.237), or the history of low-dose radiation (*p*=0.379). It was not related to the histologic type of the tumor (*p*=0.554), too (Figure 1).

**Fig. 2:**
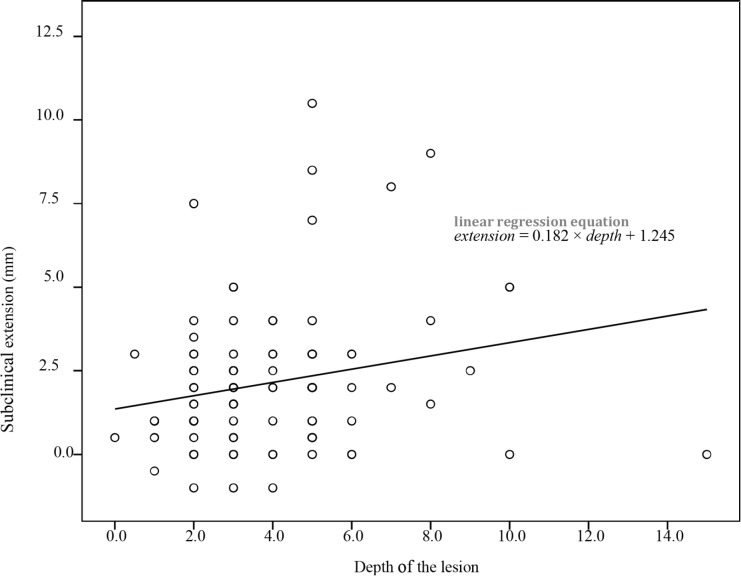
The relationship of subclinical extensions and the depth of the lesion

**Fig. 3:**
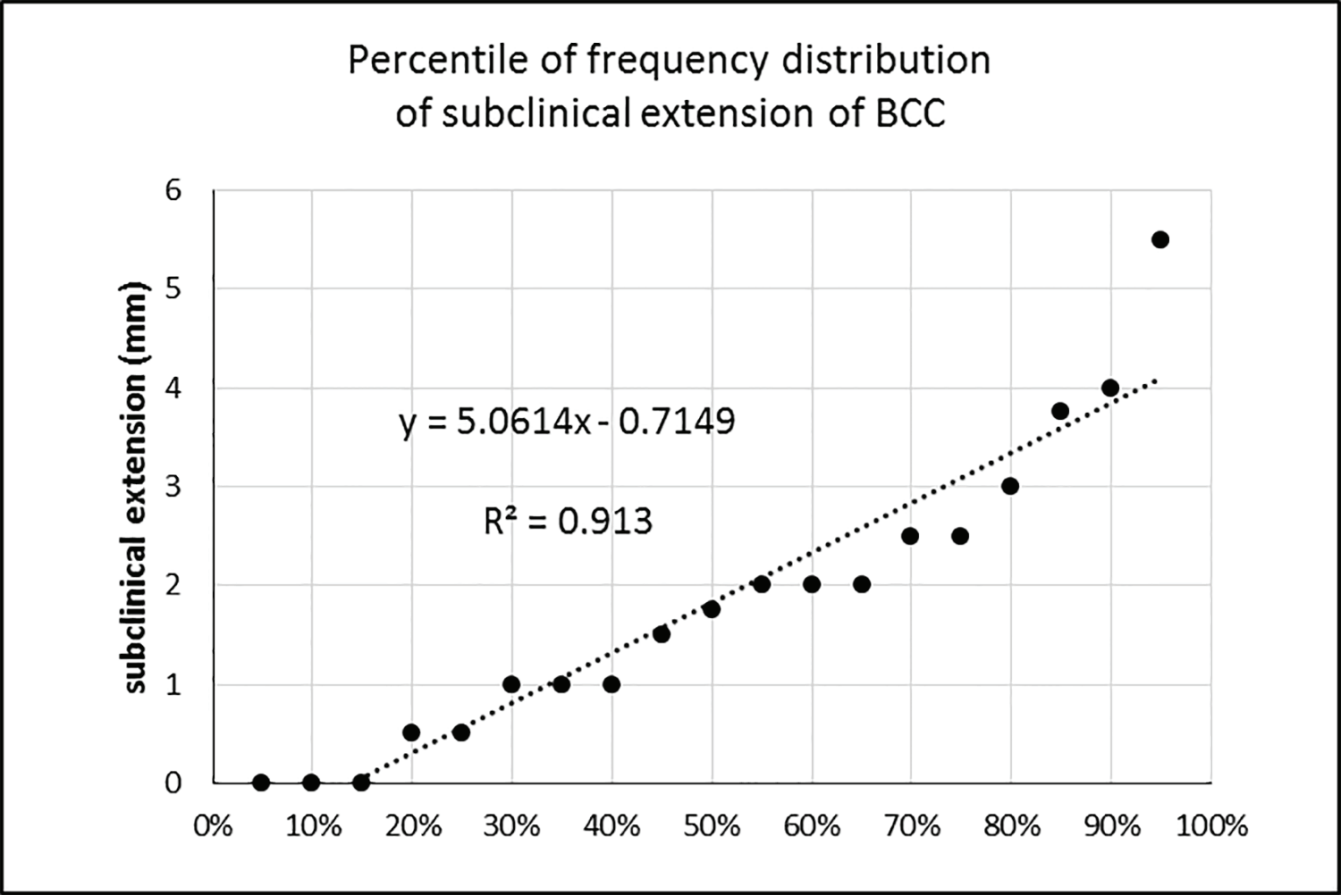
Percentile of frequency distribution of subclinical extension of BCC in different histologic types.

In a separate evaluation of the cases with positive margin involvement, the mean extent of subclinical extension in these cases was 4.3±2.45 mm that was significantly more than 1.9±1.98 mm in the cases with negative margin (*p*=0.002). There was a linear correlation between the subclinical extension and the depth of the lesion (*p*=0.033 and r=0.022) (Figure 2). It should be noted that in margin positive samples, evaluation of the subclinical extension in the involved margin was not possible and this extension was investigated in other margins of the lesion. For example, if the margin of the lesion at 9 o’clock was positive for malignancy, subclinical extension was evaluated at 3, 6 and 12 o’clock and not at 9 o’clock margin. Sixty-five percent of cases had less than 2 mm subclinical extension, 80% less than 3 mm, and 90% had less than 4 mm subclinical extension. In 95% of cases, the subclinical extension was less than 5.5 mm (Figure 3).

## DISCUSSION

In present study, 63% of patients with BCC were males, and this is consistent with previous studies.^3^^,^^16^^-^^19^ This may be due to more exposure to sunlight in male gender. In our study, the mean age of men was 64 years and mean age of women was 67 years. This difference was not statistically significant. This is also compatible with other studies.^20^^,^^21^ The most common clinical subtype of BCC is the nodular form.^19^^,^^22^^-^^24^ In present study, the most common histological types included nodular (83.6%), adenoid (9.1%), and superficial types (7.1%). This is compatible with previous studies too. 

Sun-exposed areas of the head and neck are susceptible for occurrence of BCC.^3^^,^^4^^,^^8^^,^^19^^,^^20^ In our study, the most common areas were nose (31.2%), scalp (16.1%), lower eyelid (11.8%), cheeks (10.8%), forehead (8.6%), and lower lip (5.4%). The rate of positive surgical margins in our patients was 7.8%. Overall, 92.2% of the patients showed no margin involvement. The relationship between the amount of removed healthy margin and the probability of positive margin involvement has been proven in various studies. In a study by Breuninger and Dietz, the probability of positive surgical margin following removal of a BCC with diameter less than 10 mm; if taken with 2, 3, or 5 mm margin; is reported to be 30%, 16% and 5%, respectively.^9^

In cases with positive surgical margins, the maximum extent of subclinical involvement is not appreciable. For this reason, we excluded the cases with positive surgical margins (8 cases, 7.8%) from statistical analysis of the subclinical extension. However, it is interesting to note that the mean extent of subclinical extension in margin positive specimens was greater than the margin negative specimens. This difference was statistically significant by means of student’s t Test.

In the current study, 83.0% of the specimens had subclinical extension of the tumor while in 17.0% of the specimens, no extension was observed. The minimum difference between deep and surface radius to be considered as positive subclinical extension was considered 1 mm. In Wolf and Zitelly’s study, 27% of patients had 1 mm subclinical growth while the other 73% had more than 1 mm extension.^25^ In a study by Ro *et al.*, the size of the lesion was an important factor for subclinical extension.^15^ It has been shown that BCCs with diameters greater than 2 cm have more subclinical extension, but in tumors smaller than 2 cm, there was no correlation between subclinical extension and the size of the tumor.^25^

In the present study, no positive correlation was observed between the subclinical extension of BCC and the size of the lesion (*p*=0.591, r=0.056). So, larger tumors do not require wider margin of excision. It may be due to the small number of the lesions larger than 2 cm (10 cases, 10.2%) in our study. This study demonstrated a linear correlation between subclinical extension and the depth of the lesion (*p*=0.033, r=0.022). This relationship was statistically significant using linear regression, too. It means that the lesions with deeper involvement need wider margin of resection. 

No other study has mentioned the evaluation of this relationship. However, it has been shown that male gender, larger tumor diameter, and some histological subtypes have correlation with the depth of the BCC.^26^ There is a need for further studies to prove the direct relationship between the subclinical extension and the depth of BCC lesions and investigate its clinical application. In the study by Ro *et al.*, in which Mohs micrographic surgery (MMS) was used for tumor resection, the two most common sites requiring more than one-stage surgery for eradication of the tumor were the nose and cheek. However, it cannot be considered statistically significant due to the small sample size.^15^

In the study by Malik and his colleagues on 1832 BCC patients, incomplete excision of the tumor in the nose was more likely.^27^ In the present study, we could not document a definite relationship between the site of the lesion and subclinical extension. Other studies with larger sample sizes are required to evaluate the effect of the location of the lesion on the subclinical extension. Hassanpour *et al.* and Randle, in two separate studies, have shown the relationship between the history of low-dose radiation and aggressive behavior of BCC.^28^^,^^29^

Based on the Hassanpour *et al.*’s study, the number of lesions, frequency of recurrence and aggressive types were more common in the patients with a history of low-dose radiation. However, our study did not demonstrate any relationship between low-dose radiation and subclinical extension. Larger sample sizes are required for more accurate evaluation of this relationship. The more aggressive BCCs may require more than one stage MMS to eradicate the tumor.^15^^,^^30^

In our study, there was no correlation between BCC subtypes and the subclinical extension. So it was demonstrated that margin of resection was not affected by the histologic subtype. According to the Wolf and Zitelli’s study, a 2, 3, or 4 mm margin for resection of BCCs with diameters less than 2 cm would eradicate the tumor in approximately 70%, 85%, and 95% of lesions, respectively.^25^ We found that removal of BCCs with 2, 3, and 4 mm margins lead to eradication of the tumor and its subclinical extension in 65%, 80%, and 90% of the patients, respectively. 

If we resected the tumor with 5.5 mm margin, we were sure with 95% confidence rate that we had removed the tumor and its subclinical extension area. It should be noted that we had no limitation in the tumor size for enrollment in the study. However, in our study, the size of the lesion had no effect on the extent of subclinical extension. The most important limitation of this study was its small sample size. Therefore, larger sample sizes and multi-center study with a larger number of samples are required.

In the current study, in 83% of the BCC pathology specimen, a subclinical extension was seen. No correlation was found between subclinical extension and the size or the histological subtype of BCCs. There was a direct correlation between the subclinical extension and the depth of the tumor. So, the lesions with deeper involvement required more margin of resection while it was not true for larger tumors or aggressive subtypes. According to our study, removal of BCCs with 2, 3, and 4 mm margins lead to eradication of the tumor and its subclinical extension in 65%, 80%, and 90% of the patients, respectively. Therefore, we showed that removal of the tumor with a margin of 5.5 mm would eradicate the whole lesion including the subclinical extension area with 95% confidence rate.
